# Phenylbutyric Acid Rescues Endoplasmic Reticulum Stress-Induced Suppression of APP Proteolysis and Prevents Apoptosis in Neuronal Cells

**DOI:** 10.1371/journal.pone.0009135

**Published:** 2010-02-09

**Authors:** Jesse C. Wiley, James S. Meabon, Harald Frankowski, Elise A. Smith, Leslayann C. Schecterson, Mark Bothwell, Warren C. Ladiges

**Affiliations:** 1 Department of Comparative Medicine, University of Washington, Seattle, Washington, United States of America; 2 Department of Physiology and Biophysics, University of Washington, Seattle, Washington, United States of America; University of North Dakota, United States of America

## Abstract

**Background:**

The familial and sporadic forms of Alzheimer's disease (AD) have an identical pathology with a severe disparity in the time of onset [1]. The pathological similarity suggests that epigenetic processes may phenocopy the Familial Alzheimer's disease (FAD) mutations within sporadic AD. Numerous groups have demonstrated that FAD mutations in presenilin result in ‘loss of function’ of γ-secretase mediated APP cleavage [2], [3], [4], [5]. Accordingly, ER stress is prominent within the pathologically impacted brain regions in AD patients [6] and is reported to inhibit APP trafficking through the secretory pathway [7], [8]. As the maturation of APP and the cleaving secretases requires trafficking through the secretory pathway [9], [10], [11], we *hypothesized that ER stress may block trafficking requisite for normal levels of APP cleavage and that the small molecular chaperone 4-phenylbutyrate (PBA) may rescue the proteolytic deficit.*

**Methodology/Principal Findings:**

The APP-Gal4VP16/Gal4-reporter screen was stably incorporated into neuroblastoma cells in order to assay γ-secretase mediated APP proteolysis under normal and pharmacologically induced ER stress conditions. Three unrelated pharmacological agents (tunicamycin, thapsigargin and brefeldin A) all repressed APP proteolysis in parallel with activation of unfolded protein response (UPR) signaling—a biochemical marker of ER stress. Co-treatment of the γ-secretase reporter cells with PBA blocked the repressive effects of tunicamycin and thapsigargin upon APP proteolysis, UPR activation, and apoptosis. In unstressed cells, PBA stimulated γ-secretase mediated cleavage of APP by 8–10 fold, in the absence of any significant effects upon amyloid production, by promoting APP trafficking through the secretory pathway and the stimulation of the non-pathogenic α/γ-cleavage.

**Conclusions/Significance:**

ER stress represses γ-secretase mediated APP proteolysis, which replicates some of the proteolytic deficits associated with the FAD mutations. The small molecular chaperone PBA can reverse ER stress induced effects upon APP proteolysis, trafficking and cellular viability. Pharmaceutical agents, such as PBA, that stimulate α/γ-cleavage of APP by modifying intracellular trafficking should be explored as AD therapeutics.

## Introduction

The aggregation of misfolded proteins in early compartments of the secretory pathway occurs in many neurodegenerative diseases, including Alzheimer's disease (AD) [recently reviewed [12]]. One of the primary intracellular sites in which misfolded proteins accumulate is the endoplasmic reticulum (ER). Improperly folded proteins generally fail to traffic out of the ER, as they are retained by the resident chaperone-mediated ER quality control mechanisms [13], [14], when the rate of protein synthesis exceeds the capacity of the ER to direct proper folding of the de novo proteins. Under these circumstances, elevated levels of unfolded proteins result in a phenomenon known as ER stress, which initiates a set of events known as the unfolded protein response (UPR). UPR signaling is a multifaceted cascade designed to either restore ER homeostasis or terminate the cell [13], [14]. Consequently, there is transcriptional up-regulation of specific chaperones, transcription factors, and a regulated interruption of some classes of translation. The identification of active UPR signaling in AD patients, specifically within the pathologically affected brain regions, suggests that ER stress contributes to the pathological progression in AD [15]. Recent reports show that beta-amyloid precursor protein (APP) trafficking through the secretory pathway is impaired under ER stress conditions, suggesting that APP is retained within the ER, or early components of the secretory pathway [7], [8]. The link between AD and ER stress likely involves trafficking defects, as altered trafficking of APP and the cleaving secretases would be predicted to directly impact APP cleavage—a central component of AD pathogenesis.

APP and the cleaving secretases traffic and mature through the secretory pathway. The activity of the secretases is dependent upon their glycosylation and proteolytic maturation, which occurs in later components of the secretory pathway, most notably within the Golgi[16], [17]. The cleavage of APP by α-secretase is mediated by members of the metalloproteinase family [18], with TACE and ADAM10 being the predominant members [19]. TACE and ADAM10 are activated by the furin-mediated proteolytic release of their regulatory domains, which occurs in the Golgi complex [16]. The pro-amyloidogenic processing of APP is initiated by β-secretase cleavage in the extracellular domain by the atypical aspartyl protease BACE [20]. The proteolytic maturation of BACE also requires processing within the Golgi complex [21], [22]. Furthermore, the complex formation and activation of the heterotetrameric γ-secretase complex requires maturation in the Golgi [23], [24], in which presenilin-1 (PS1) and Nicastrin are both glycosylated and the holo-form of presenilin is cleaved into the amino- and carboxy-terminal fragments (NTF and CTF respectively) required for γ-secretase activity [9]. Consequently, inducing a state of ER stress, which can subsequently halt the passage of proteins through the ER, could directly impact the secretase mediated proteolytic processing of APP at multiple levels.

The possibility of ER stress playing a role in AD pathogenesis is particularly interesting in light of the discovery of bioactive and bioavailable small molecules which act like chaperones in promoting protein folding and subsequent trafficking through the secretory pathway. These small molecules are referred to as small molecular chaperones as they are believed to decrease the energy barrier between the intermittent transition-states that occur as proteins fold into their native conformation [25]. Small molecular chaperones were originally developed to promote cystic fibrosis transmembrane conductance regulator (CFTR) trafficking, and one member of the family, phenylbutyric acid (PBA) is already FDA approved for treatment of urea cycle disorder. PBA, and other members of the small molecule chaperone family, consistently decrease the levels of UPR signaling, supporting the protective effects of these compounds against ER stress [26]. Significantly, PBA is also blood-brain barrier permeable [27], making it an exciting candidate therapeutic for neuropathological conditions.

We hypothesized that induction of ER stress would decrease the secretase-mediated generation of the APP intracellular domain (AICD) associated with APP nuclear signaling. However, the AICD is difficult to quantitate by standard biochemical means owing to its small size and its remarkable instability [28]. Consequently, in order to test this hypothesis, we generated a strain of stably transformed N2a neuroblastoma cells containing the Gal4-luciferaseEGFP γ-secretase reporter system we recently engineered. In this genetic reporter system, γ-secretase mediated cleavage of APP fused in frame to the yeast-viral hybrid transcription factor Gal4-VP16 (referred to as APPGV16) activates the Gal4-luciferaseEGFP reporter gene. Using this system we demonstrate that pharmacological agents that induce ER stress consistently decrease γ-secretase mediated cleavage of APP. Furthermore, we show that co-treatment with the small molecule chaperone PBA rectifies the proteolytic deficit, stimulates AICD production, promotes trafficking and protects against ER stress induced apoptosis.

## Materials and Methods

### Chemicals and Antibodies

The selection reagents zeomycin (Invitrogen) and G418 (Cellgro) were used at 200 µg/ml and 500 µg/ml respectively. Tunicamycin (Calbiochem) was dissolved in DMSO at a stock concentration 10 mg/ml. Thapsigargin (Sigma) was dissolved in DMSO at 0.5 mM concentration. Brefeldin A (MP Biomedicals) was suspended in DMSO at 10 mg/ml. The phenylbutyric acid (4-PBA, Calbiochem) was dissolved in filtered sterile water at a 1M stock concentration. The VP16 antibody (Sigma) used for subcellular localization of APPGV16 was diluted at 1:500 for immunohistochemistry. The donkey anti-rabbit Alexa 546 (Molecular Probes) antibody secondary was employed with the VP16 antibody in subcellular localization studies at a 1:5000 dilution. The eIF2α (Cell Signaling), phoshpo-eIF2α (Cell Signaling), and phospho-PERK antibody (Cell Signaling) were diluted for western blot at 1:1000. The Gal4 antibody (Calbiochem) used for identification of APPGV16 protein and its proteolytic fragments by western blotting was diluted at 1:500. The phospho-JNK and phospho-p38 antibodies (Cell Signaling) were both used at 1∶1000. Luciferin (Biosynth) was reconstituted at 1 mM for the luciferase assays. Galacton (Tropix) and Emerald Amp (Tropix) were used for the β-galactosidase normalization assays.

### Vector Construction

**Figure 1:**
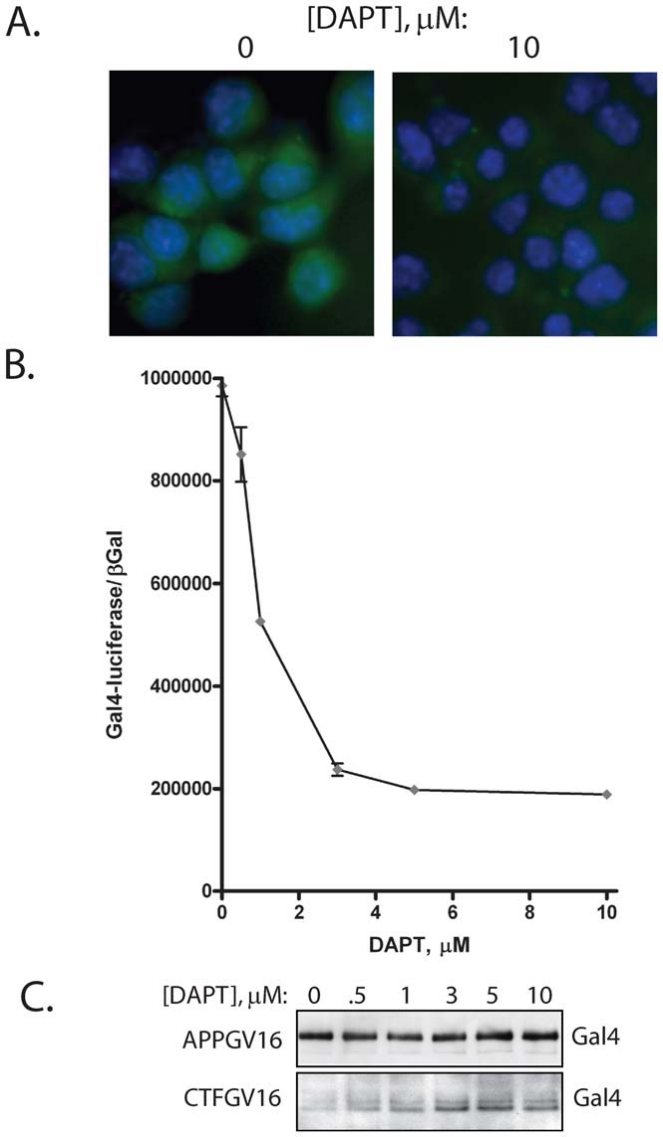
γ-secretase dependence of the APPGV16/Gal4-luciferaseEGFP/EF1- NLS-βGal reporter cells. The N2a EF1-APPGV16/Gal4-luciferaseEGFP/EF1-NLS-βGal stable cells (NAG cells) have γ-secretase dependent EGFP expression, as DAPT eliminates the majority of EGFP fluorescence (A). The Gal4-luciferase moiety of the reporter is also γ-secretase dependent as titrated DAPT diminishes luciferase activity (B). The decrease in luciferase activity correlates with elevated CTFGV16 protein levels across the DAPT titration (C).

The tricistronic reporter construct was developed by fusing pieces from numerous different expression vectors. The Gal4-luciferase vector (pFRluc, Stratagene) was used as an initial template for construction of the reporter vector. Primers were designed against the Gal4-luciferase operon in which the SalI-BglII restriction site sequences were added to the 5′ end of the 5′ primer. The StyI site was added to the 5′ end of the 3′ primer against Gal4-luciferase and the stop codon was deleted. High fidelity PCR amplification of the Gal4-luciferase sequence containing the new restriction sites was completed, and the SalI-StyI Gal4-luciferase fragment was cloned into the SalI-XbaI sites in CMV-EGFP. CMV-EGFP was generated by amplifying the EGFP sequence from pEGFP (Clontech) and inserting XbaI at the 5′ end and XmaI at the 3′ end of the coding sequence. This was cloned into the XbaI-XmaI sites in GFPxlt (provided by Dr Randall Moon), following site-directed mutagenesis to insert XmaI in between the 3′ end of the GFP coding sequence and the polyA sequence. Subsequently, a BglII site was inserted 3′ to the polyA sequence for later subcloning events. The CMV promoter in CMV-EGFP was deleted by inserting Gal4-luciferase into the vector, as the SalI site in CMV-EGFP was 5′ to the CMV promoter. The cloning of Gal4-luciferase in frame with EGFP resulted in the luciferase-EGFP fusion reporter driven exclusively by the Gal4 promoter. In order to normalize the Gal4-transactivation data, a constitutively expressing nuclear targeted β-galactosidase cassette was inserted into the reporter vector. The pNeoZTK2 murine gene targeting vector (provided by Dr Richard Palmiter) was used as a template. PCR primers were designed against the NLS-βGal portion of the targeting vector, with a NheI site incorporated into the 5′ portion of the 5′ primer and a HindIII site added to the 5′ end of the 3′ primer. The NLS-βGal coding region was amplified by high fidelity PCR, and the NheI-HindIII fragment was subcloned into pcDNA3.1Zeo (Invitrogen). The CMV promoter was removed and replaced by inserting the elongation factor 1α promoter amplified off of the pCEFL expression vector with NruI and NheI sites contained in the 5′ and 3′ primers respectively. This resulted in the generation of EF1-NLS-βGal. Two BglII sites were identified and mapped in EF1-NLS-βGal. The one adjacent to the EF1-NLS-βGal coding region was eliminated by linearizing the plasmid through a partial digest and blunt ending the vector and religating the blunt ends. The second BglII site was used to subclone the BglII-Gal4-luciferaseEGFP-BglII fragment containing the associated polyA site. This final step resulted in the tricistronic Gal4-luciferaseEGFP/EF1-NLS-βGal vector that was used to generate the stable N2a reporter cells (Figure 1).

### Generation of APPGV16/Gal4-LuciferaseEGFP/EF1-NLS-βGal Stable γ-Secretase Reporter Cells

The EF1-APPGV16 expression vector described previously [5] contains a neomycin resistance element. The Gal4-luciferaseEGFP/EF1-NLS-βGal construct contains a zeomycin resistance element. 10 µg of EF1-APPGV16 and Gal4-luciferaseEGFP/EF1-NLS-βGal were transiently co-transfected into a confluent 10 cm plate of naïve neuroblastoma N2a cells. Two days post-transfection the N2a cells were passaged and placed under dual selection with 500 µg/ml G418 and 200 µg/ml Zeocin (Invitrogen). These cells were maintained under selection for approximately three weeks, at which point individual clones were isolated and cultured in single wells of a 24 well plate. Once the clones were confluent, they where split, passaged, and screened for DAPT-sensitive luciferase activity and constitutive βGal activity by standard luminometric assays. The clone used in this study was selected due to high levels of γ-secretase dependent (DAPT sensitive) luciferase activity and consistent βGal activity. The cells were expanded and continuously grown under selection. The **N**2a **A**PPGV16/**G**al4-luciferaseEGFP/EF1-NLS-βGal (NAG) cells were employed throughout the work presented here.

### Cell Culture Conditions

The NAG cells were grown in DMEM supplemented with 10% fetal bovine serum (FBS) (Hyclone) at 37C in 10% CO_2_. The stock plates were maintained with 500 µg/ml G418 and 200 µg/ml Zeocin to maintain stability of the genetic elements. The experiments were performed when the NAG cells were between 50 and 75% confluent. Comparisons between the Gal4-transactivation levels and protein levels were done within the same experiment, unless otherwise indicated, wherein experimental conditions were staged in quadruplicate. Three wells were used per conditions for Gal4-transactivation assays, and 1 well was utilized for western blotting. In the experiments employing immunohistochemical approaches, the NAG cells were plated on 18mm coverslips coated with poly-D-lysine in 12 well plates. The transactivation assays and western blots were performed 24 hours following treatment unless otherwise indicated.

### Western Blots and Transactivation Assays

The NAG cells were passaged from 60–80% confluent stock plates, and arrayed on 24 well plates for transactivation assays and western blotting. The transactivation assays were performed by lysing the NAG cells in 0.1% TX-100 lysis solution and were frozen at −80C to ensure complete lysis. The Gal4-luciferase and βGal luminometric assays were performed as previously described [5], [29] employing a Berthold EG&G MicroLumat LB 96V luminometer. The output from the luminometric assays were point to point normalized, dividing the raw luminometric output from the luciferase assay by the βGal value for each sample. This data is plotted as Gal4-luciferase/βGal in the figures to denote that the raw luciferase data is normalized to constitutive reporter expression. The transactivation assays were performed in triplicate, and the standard deviation was calculated based upon the normalized value for each sample in each condition. The lysates for western blotting were generated using standard 1XRIPA buffer supplemented with 1X Protease Inhibitor Cocktail (Sigma). The lysates were rotated for 10 minutes at 4C, and spun for 30 minutes at 10,000g at 4C. The supernatant was mixed with 5X loading buffer and the blots were run using the NuPage Novex 4–12% gradient Bis-Tris gels (Invitrogen) with MOPS running buffer (Invitrogen). The blots were transferred to 0.2 µM nitrocellulose and all primary antibody incubations were performed at 4C overnight.

### Immunohistochemistry

NAG cells were arrayed on poly-D-lysine coated coverslips in 12 well plates. The cells were treated as indicated and probed with the designed antibodies. The images were collected on either a Zeiss Axioskop 2 fluorescent microscope (Figure S1), or on a Leica SP1 laser scanning confocal microscope. The images were taken adjusting all settings to the untreated condition. All the settings were held constant for the remainder of the images to maintain accurate relative fluorescence between conditions. The primary antibodies were previously discussed. APPGV16 was visualized using the rabbit VP16 antibody and the Donkey anti-rabbit Alexa 546 (Molecular Probes) secondary. DAPI (Vectashield) or Hoescht nuclear stains were used to resolve the nuclei of the cells. The pycnotic nuclei were identified in the apoptosis assays by counting the number of cells with either condensed or fragmented nuclei relative to the total number of cells in each condition by an observer blinded to the identity of the experimental conditions. No less than 1000 cells were counted per condition.

## Results

### Stably Transfected Neuroblastoma Cells Can Be Used to Measure γ-Secretase Activity

The neuroblastoma N2a APPGV16/Gal4-luciferaseEGFP/EF1-NLS-βGal (NAG) cells were generated as described in the Materials and Methods section. The NAG cells employ a Gal4-driven luciferase-EGFP fusion reporter which quantitatively measures γ-secretase cleavage of APPGV16. The APPGV16/Gal4-reporter system was employed successfully as independent vector components in transiently transfected cells[5], [29]; yet this is the first report of the reconstruction of this assay system into stably transfected neuroblastoma N2a cells. As a novel model, we validated that the Gal4-reporter output corresponds to γ-secretase activity. We tested both the luciferase and the EGFP components of the Gal4-reporter system with the addition of the γ-secretase inhibitor DAPT. The NAG cells treated with DAPT for 24 hours demonstrated almost no EGFP signal, which was robust in the untreated cells (Figure 1A). This validated the γ-secretase dependence of the Gal4-EGFP output. Quantitative measures of the luciferase output in NAG cells treated with titrated concentrations of DAPT for 24 hours were employed to determine the γ-secretase dependence of the raw luminometric output. These values were normalized to βGal activity. Consistent with the EGFP results, the luciferase activity decreased monotonically with increasing DAPT concentrations, with a maximal inhibition of 80% of Gal4-reporter activity (Figure 1B). The IC50 was approximately 1 µM DAPT, consistent with previous reports of DAPT inhibition of γ-secretase [30], [31]. DAPT did not impact the normalization values in the βGal assays, ruling out toxic or non-specific transcriptional effects. As occurs with endogenous APP, DAPT treatment resulted in elevated levels of the α- and β-secretase derived APP carboxy-terminal fragments (CTFα and CTFβ), with CTFα being the predominant proteolytic species observed (Figure 1C). The holoAPPGV16 levels did not change across the DAPT titration, ruling out APPGV16 down-regulation, or variations in protein loading, contributing to the DAPT mediated decrease in reporter activity. Consequently, the Gal4-reporter system, stably incorporated into the NAG cells, is a valid measure of γ-secretase mediated proteolytic processing of APPGV16.

### Endoplasmic Reticulum (ER) Stress Induction Results in Impaired APP γ-Proteolysis and Induction of Apoptotic Signaling (Figures 2–4)

**Figure 2:**
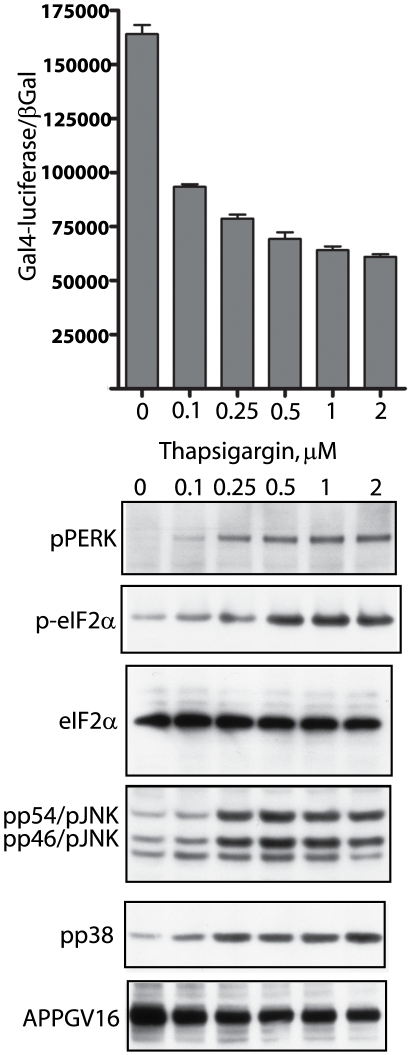
Thapsigargin inhibits APP proteolysis and induces ER stress UPR signaling. Increasing doses of thapsigargin inhibits γ-secretase cleavage of APPGV16 (top graph). Titrated doses of thapsigargin induced graded increases in UPR stress signaling through phospho-PERK (pPERK blot) and phospho-eIF2α (p-eIF2 α), beginning at 0.1 µM and reaching a plateau at 0.5 µM thapsigargin. The increases in UPR signaling correlate with the levels of phospho-p38 (pp38 blot) and phospho-JNK (pp54/JNK, pp46JNK blot), as maximal stimulation is observed in the same concentration range. Total protein expression levels remain unchanged, as eIF2α and APPGV16 protein levels were consistent across treatment conditions.

**Figure 3 pone-0009135-g003:**
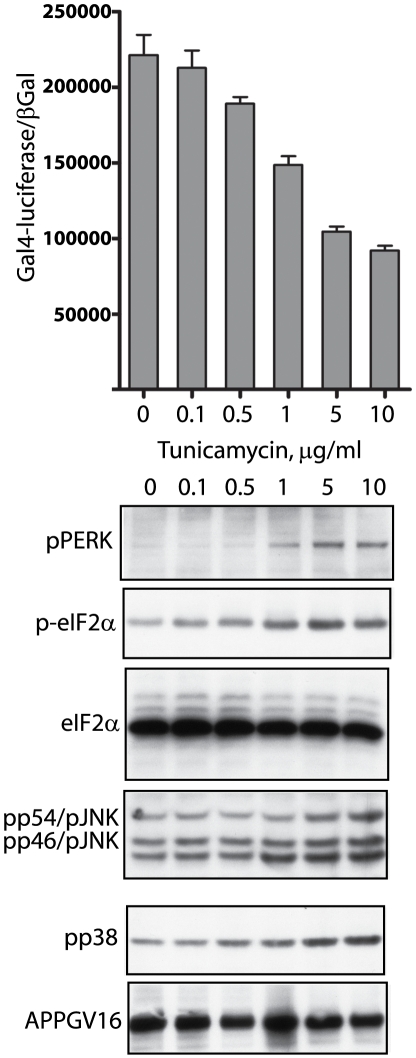
Tunicamycin inhibits APP proteolysis and induces ER stress UPR signaling. The NAG cells were treated with titrated doses of the N-glycosylation inhibitor tunicamycin, which inhibits γ-secretase cleavage of APPGV16 (top graph). Increasing doses of tunicamycin induced elevated levels UPR activation, demonstrated by elevated levels of phospho-PERK (pPERK blot) and phospho-eIF2α (p-eIF2 α) levels. Tunicamycin elevated phospho-p38 (pp38 blot) and phospho-JNK (pp54/JNK, pp46JNK blot) levels in parallel with increased UPR signaling and decreased APPGV16 proteolysis.

**Figure 4:**
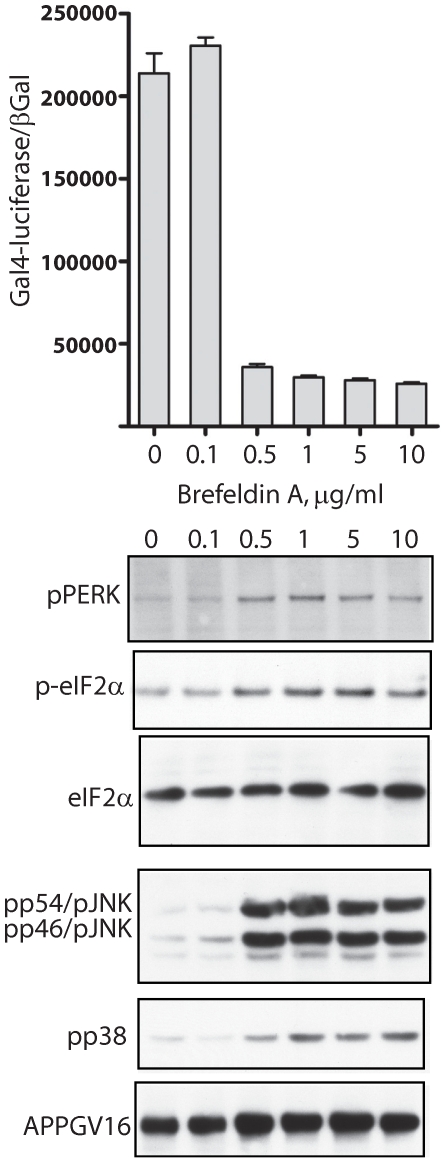
Brefeldin A inhibits APP proteolysis and induces ER stress UPR signaling. Brefeldin A inhibited γ-secretase cleavage of APPGV16 (top graph). Western blots show that 0.5 µg/ml Brefeldin A induces activation of PERK (pPERK blot) and eIF2α (p-eIF2 α). Increases in phospho-p38 (pp38 blot) and phospho-JNK (pp54/JNK, pp46JNK blot) levels are also observed at the same concentration of BFA which fosters increased UPR signaling and diminished γ-secretase dependent APPGV16 cleavage.

As already mentioned, ER stress is implicated in AD pathogenesis; however, how it impacts γ-secretase processing of APP is relatively unexplored. ER stress effects upon APP proteolysis were measured in NAG cells treated with titrated concentrations of three different compounds: tunicamycin, thapsigargin and brefeldin A (BFA). Tunicamycin is a well known inhibitor of N-glycosylation, preventing N-glycan maturation upon transmembrane proteins within the secretory pathway [13]. Thapsigargin is a sarcoplasmic/endoplasmic reticulum calcium ATPase (SERCA) pump inhibitor, blocking Ca^2+^-reuptake into the ER, and impairing calcium dependent chaperone function [32]. BFA blocks trafficking through the Golgi by targeting the ADP ribosylation factor guanosine triphosphate hydrolase (Arf-GTPase), which results in the dissolution of the Golgi apparatus [33]. The NAG cells were treated for 18–20 hours and employed in transactivation assays, to measure gamma-secretase mediated APPGV16 cleavage, and in western blots examining ER stress markers. As markers for ER stress induction, we examined PKR-like ER kinase (PERK) and eukaryotic translation initiation factor 2α (eIF2α) phosphorylation—two critical components of UPR signaling. As ER stress culminates in the activation of the pro-apoptotic kinases JNK and p38 [34], we included phospho-JNK and phospho-p38 in our examination.

In the NAG cells treated with thapsigargin, there is an inverse relationship between APP proteolysis and the activation of UPR pathways. Gal4-reporter activity decreased with increasing concentrations of thapsigargin (Figure 2, top). Conversely, thapsigargin induced phospho-PERK, phospho-eIF2α and activation of p38 and JNK (Figure 2) between 0.1 µM and 0.5 µM. Maximal stimulation of each UPR pathway occurs by 0.5 µM thapsigargin. Concordantly, thapsigargin maximally inhibits APP proteolysis by 0.5 µM, suggesting that repression of APP proteolysis occurs coordinately with the induction of ER stress. Maximal repression of Gal4-reporter activity by thapsigargin results in a decrease of 63 percent, less than observed with DAPT, suggesting that ER stress conditions permit low levels of γ-secretase mediated APPGV16 proteolysis. Low level stress also attenuates γ-secretase mediated APP proteolysis, as the addition of 0.1 µM thapsigargin, which correlates with slight increases in the UPR markers, decreases Gal4-reporter activity.

Similarly, tunicamycin treatment induced ER stress and decreased γ-secretase mediated APP proteolysis (Figure 3). Tunicamycin inhibited 59 percent of Gal4-reporter activity at the highest titrated dose. Thapsigargin and tunicamycin inhibit APP proteolysis to a similar degree, suggesting that a common mechanism may be involved. Additionally, Gal4-reporter output decreases in a linear inverse relationship with the appearance of UPR markers. The concentration of tunicamycin which induces detectable levels of phospho-PERK and phospho-eIF2α (1 µg/ml) inhibits γ-secretase mediated APP proteolysis by approximately 50 percent. Repression of APP proteolysis and stimulation of UPR signaling are both maximal at 5 µg/ml tunicamycin. The tight coupling of APPGV16 proteolytic repression and ER stress induction, following tunicamycin treatment, supports a direct relationship between ER stress and APP proteolysis.

Unlike thapsigargin and tunicamycin, the dose-response curve for both UPR activation and APP proteolytic repression is abrupt and non-linear with BFA treatment (Figure 4). Maximal repression of APPGV16 cleavage occurs concordantly with activation of the UPR markers. APPGV16 protein levels remain even, or increase slightly, with BFA treatment—demonstrating that change in APPGV16 protein expression is not responsible for the repressive effects observed. The inverse relationship between APP proteolysis and UPR activation subsequent to BFA treatment is consistent with the effects of tunicamycin and thapsigargin. However, BFA more potently inhibits APPGV16 proteolysis than either thapsigargin or tunicamycin—decreasing Gal4-reporter activity by 88 percent. The degree of proteolytic repression with BFA is similar to that observed with DAPT, demonstrating the essential role of ER to Golgi trafficking upon secretase mediated APP processing.

All three secretases (α,β, and γ) undergo glycosylation mediated maturation through the secretory pathway. As APP proteolytic processing is a sequential process, the repression of any individual secretase would inhibit release of the AICD. Consequently, we examined the effects of ER stress induction upon two other proteolytic fragments directly: C99 and the AICD. DNA vectors encoding each protein fragment fused to Gal4VP16 were transfected into naïve N2a cells. The transfected cells were treated with tunicamycin, thapsigargin or BFA. Consistent with previous reports showing that γ-secretase activity requires trafficking into later compartment of the secretory pathway [9], all three ER stress inducing compounds block γ-secretase mediated cleavage of C99GV16 to varying degrees. Thapsigargin and BFA induced statistically significant levels of APP proteolytic repression (Figure S2). Confirming that ER stress induction does not influence the genetic reporter system, there is no significant or systematic shifts in C50GV16 (the γ-secretase cleaved AICD-GV16 portion of APPGV16) driven Gal4-reporter activity in the presence of the stress inducing compounds.

### Small Molecular Chaperone PBA Results in Restored AICD Production under Stress Conditions

**Figure 5:**
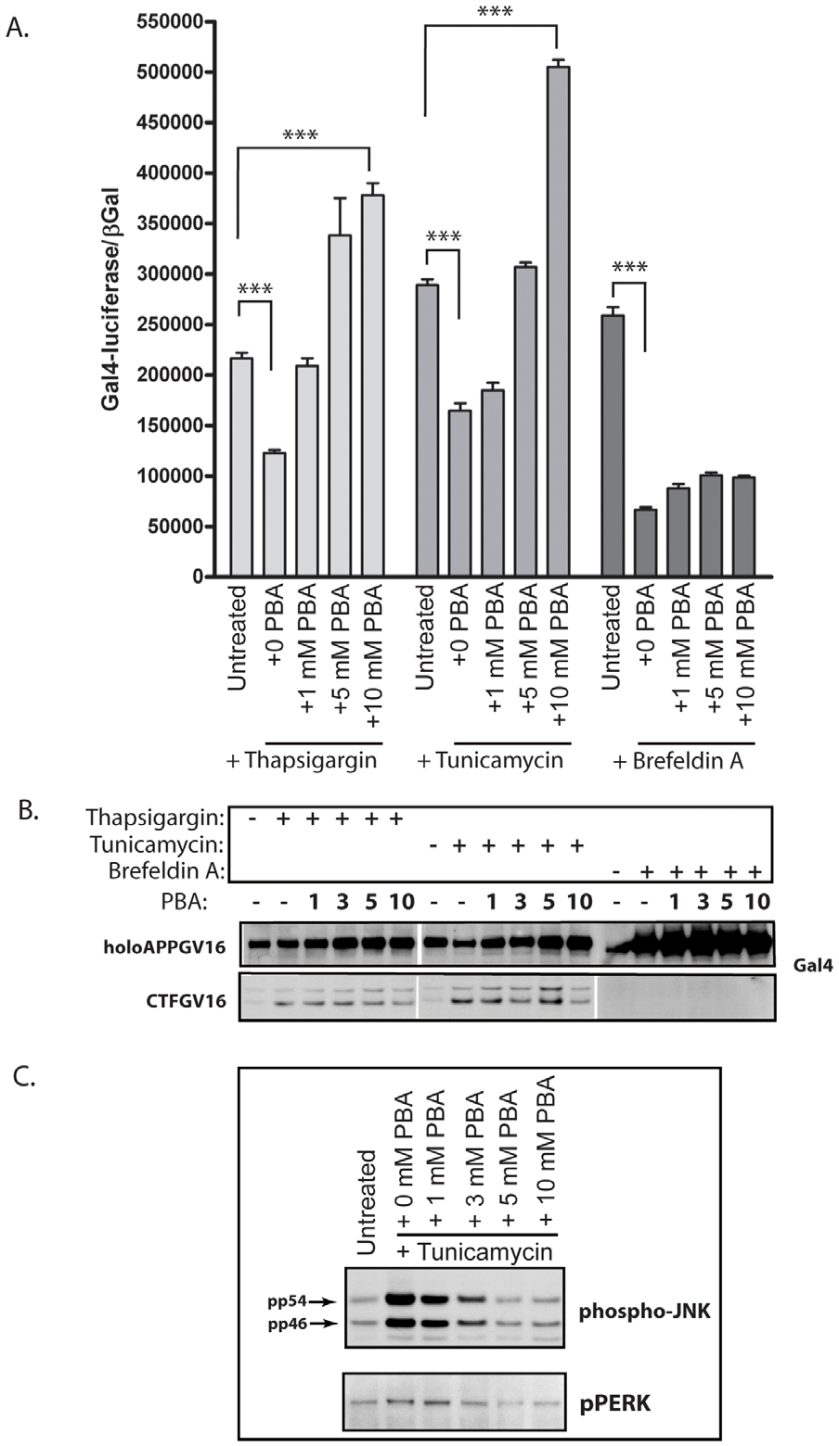
Small molecular chaperone 4-phenylbutyric acid (PBA) rescues APP proteolysis from thapsigargin and tunicamycin treatment. NAG cells either remained untreated, or were treated with thapsigargin (0.25 µg/ml), tunicamycin (5 µg/ml), or brefeldin A (5 µg/ml) alone, or in conjunction with titrated values of PBA, for 24 hours. Gal4-luciferase measures were normalized to constitutive NLS-βGal expression. Treatment with each stress inducing compound resulted in significant decreases in APPGV16 proteolysis (p<0.0002, unpaired t-test) (A). Co-treatment with PBA rescued the decrease in APPGV16 proteolysis, promoting cleavage beyond that observed in untreated cells in the presence of thapsigargin or tunicamycin (p<0.0001, t-test). PBA had no significant effect upon APPGV16 proteolysis in the NAG cells treated with brefeldin A. The effects of the stress inducing compounds and PBA upon APPGV16 and CTFGV16 protein levels were examined in parallel experiments (B). Thapsigargin and tunicamycin treatment resulted in elevated CTFGV16 levels. Co-treatment with PBA resulted in minor decreases in CTFGV16 levels by the titration endpoint. No CTFGV16 proteolytic fragments were observed in the brefeldin A treated NAG cells. PBA inhibited UPR signaling (phospho-JNK and phospho-PERK) induced by 5 µg/ml tunicamycin (C).

Small molecular chaperones are a class of compounds which facilitate protein folding and subsequent trafficking through the secretory pathway [25]. Numerous reports confirm that PBA, one member of the small molecular chaperone class, relieves ER stress and UPR signaling [35], [36], [37]. Consequently, we employed PBA to test whether small molecular chaperones could rectify APP proteolysis under ER stress conditions. NAG cells were grown in the presence or absence of all three ER stress inducing compounds and treated with titrated levels of PBA from 0 to 10 mM for 24 hours. As previously observed, the addition of thapsigargin, tunicamycin or BFA induced a statistically significant decrease in the levels of γ-secretase mediated APP cleavage (Figure 5A). Co-treatment with PBA eliminated the repression of APP proteolysis associated with thapsigargin and tunicamycin treatment (Figure 5A), increasing Gal4-reporter activity above basal levels by 10 mM. However, PBA had no effect upon BFA induced decreases in γ-secretase mediated APPGV16 processing. Since BFA treatment results in Golgi dissolution, and eliminates the possibility of trafficking through the secretory pathway, the lack of any PBA effect in BFA treated cells strongly suggests a role for PBA in stimulating trafficking under stress conditions. Neither the decrease in APPGV16 proteolysis brought about by the ER stress inducing compounds, nor the PBA mediated increase in APPGV16 cleavage, is due to changes in APPGV16 protein levels, as there is no correlation observed between expression level and Gal4-reporter activity (Figure 5B).

The α-, β-, and γ-secretases undergo maturation by proteolysis and glycosylation as they travel through the secretory pathway [9], [16], [17], [22]. Consequently, the activity of all three secretases could be inhibited under ER stress inducing conditions due to diminished trafficking through the secretory pathway. In order to assess whether the activity of all three classes of secretases are inhibited under stress conditions, we examined the levels of the CTF-GV16 species in the various treatment conditions. Consistent with the interpretation that ER stress induces inhibition of the γ-secretase complex, elevated levels of CTF-GV16 were observed following treatment with both thapsigargin and tunicamycin (Figure 5B). This suggests that thapsigargin and tunicamycin more significantly impact γ-secretase processing than either α- or β-secretase processing, but does not eliminate the possibility that stress induction may attenuate α- or β-secretases to a lesser degree. Interestingly, BFA elicits dramatic increases in the quantity of holoAPPGV16 and completely attenuates production of the CTF-GV16 species—suggesting that BFA inhibits APPGV16 processing at multiple levels (Figure 5B). BFA may block the maturation of all three secretases, by trapping them in the ER, and preventing their activation in subsequent components of the secretory pathway. Consistently, as PBA enhances APPGV16 cleavage, the level of CTF-GV16 decreases. However, some caution is necessary in interpreting these results as PBA may increase all three classes of secretase activity concomitantly.

In order to assess whether PBA induced APP proteolysis correlates with decreases in ER stress, PBA was titrated onto tunicamycin treated cells. As previously shown, both phospho-JNK and phospho-PERK levels increase with tunicamycin treatment (Figure 5C). However, co-treatment with PBA decreases both phospho-JNK and phospho-PERK levels (Figure 5C). This suggests that PBA mediated rectification of APPGV16 proteolysis occurs concurrently with decreases in UPR signaling. The concomitant increase in APP proteolysis and decrease in UPR markers, argues that stimulation of APP proteolysis occurs due to changes in ER stress related processes following PBA treatment.

### PBA Promotes APPGV16 Trafficking

**Figure 6:**
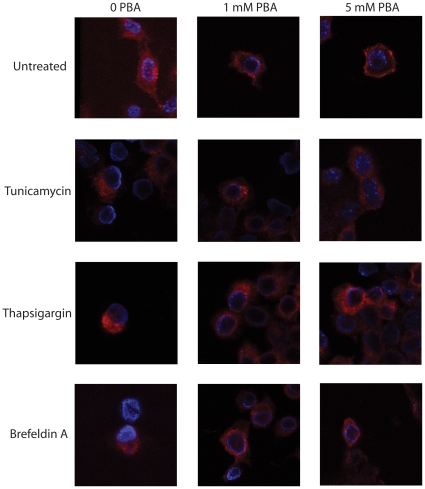
PBA stimulates trafficking out of the intracellular organelles. NAG cells were untreated, or treated with thapsigargin (0.25 µM), tunicamycin (5 µg/ml), or brefeldin A (5 µg/ml) in the presence or absence of PBA for 24 hours. The cells were stained with the VP16 antibody (red) and counterstained with Hoescht (blue) to localize the nuclei. Confocal imaging was performed to examine protein localization. In untreated cells, APPGV16 localized throughout the cytosol with slight peri-nuclear aggregations observed, consistent with the ER localization of de novo membrane proteins. PBA treatment promoted migration of APPGV16 toward the plasma membrane (top row). In tunicamycin, thapsigargin and brefeldin A treated cells, APPGV16 localized to the ER-like perinuclear region (left column, lower three images). Upon the addition of 1 mM or 5 mM PBA, APPGV16 localization shifted away from the nucleus in tunicamycin and thapsigargin treated NAG cells. In contrast, PBA had no effect upon the localization of APPGV16 in the brefeldin A treated cells.

Previous reports suggest that ER stress induction may alter APP trafficking [7], [8]. APPGV16 localization was examined in the NAG cells under non-stress inducing conditions, or in the presence of thapsigargin, tunicamycin or BFA. In the untreated cells, APPGV16 localized throughout the cell, with limited peri-nuclear aggregation, consistent with trafficking through the early secretory pathway. Treatment with all three stress-inducing agents promoted accumulation in intracellular sites that probably represent ER localization. In cells treated with BFA, the peri-nuclear aggregation was more robust, with no APPGV16 observed beyond the peri-nuclear region (Figure S1). In order to assess whether the small molecular chaperone PBA rectifies trafficking under ER stress-inducing conditions, NAG cells were treated with each of the ER stress-inducing pharmacological agents in the presence or absence of PBA. The cells were examined by thin-section laser scanning confocal microscopy. In the untreated cells, APPGV16 distributed ubiquitously throughout the cell, as previously observed. With increasing concentrations of PBA, the localization pattern shifted toward the outer region of the cell, consistent with a predominantly plasma membrane localization by 5 mM PBA (Figure 6, top row). The NAG cells treated with either tunicamycin or thapsigargin had restricted APPGV16 localization, accumulating in the peri-nuclear region, consistent with the data in Figure S1. However, in the presence of PBA, the localization of APPGV16 is more evenly distributed throughout the cell, appearing similar to basal conditions by 5 mM PBA (Figure 6, second and third rows). Discordantly, in the NAG cells treated with BFA, APPGV16 localized to the peri-nuclear region, and was unaffected by PBA (Figure 6, bottom row). These data suggest that there is a strong correlation between APPGV16 localization and proteolytic processing in the different treatment conditions.

### PBA Stimulates APP Proteolysis

**Figure 7:**
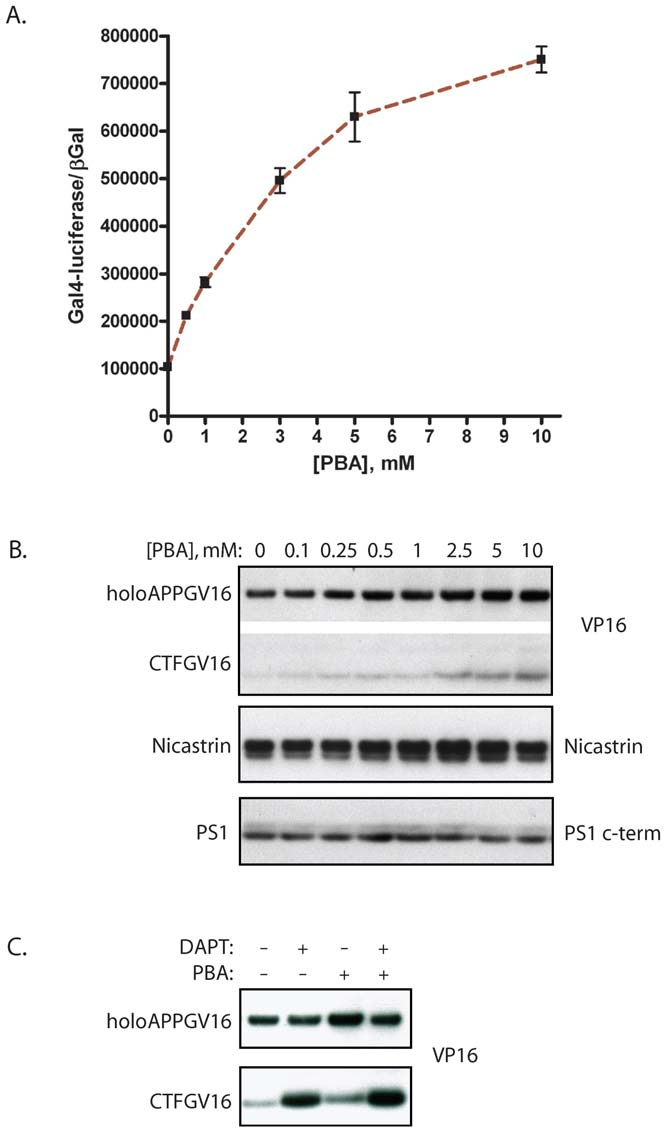
PBA stimulates secretase-mediated APP cleavage. The NAG cells were treated with titrated concentrations of PBA for 24 hours. PBA elicited elevated levels of Gal4-reporter reporter transactivation in a concentration dependent fashion (p<0.0001; student t-test 0 versus 10 mM PBA) (A). PBA effects upon APPGV16, PS1 and Nicastrin expression levels were examined in the NAG cells 24 hours post-treatment. APP-Gal4VP16 levels increased slightly across the PBA titration. The CTFGV16 proteolytic fragment, whose molecular weight corresponds to the α-cleavage product of APPGV16, increased substantially across the PBA titration (B, top blot). In contrast, no increases in Nicastrin or PS1 levels were observed (B, middle and bottom blots). CTFGV16 increased in NAG cells treated with PBA and DAPT. Co-treatment with DAPT and PBA increased CTFGV16 protein levels in an additive manner, consistent with PBA stimulating CTF-GV16 production (C).

The PBA mediated stimulation of APP proteolytic processing under ER stress conditions, and the changes in subcellular localization, suggests that PBA may promote γ-secretase mediated cleavage of APP under basal conditions. In order to assess what effects PBA has upon APP cleavage in non-stress related conditions, PBA was titrated onto NAG cells in the absence of ER stress inducing compounds. PBA treatment stimulated a dose-dependent increase in APP proteolysis, reaching a near 8-fold increase by 10 mM (Figure 7A). Higher concentrations of PBA elicited no additional increase in Gal4-reporter activity (data not shown). The PBA induced stimulation of γ-secretase mediated APPGV16 proteolysis was observed in three separate experiments, each demonstrating a similar magnitude of effect. There was no effect of PBA upon constitutive βGal expression, ruling out non-specific transcriptional effects contributing to the observed stimulation.

In parallel experiments, gradual titrations of PBA were performed to assess the effects of PBA upon APPGV16 and mature γ-secretase levels. Across the PBA titration, APPGV16 protein levels increased, but not proportionally to the observed increases in Gal4-reporter output (Figure 7B). Additionally, the levels of CTF-GV16 rose appreciably with PBA treatment, suggesting that PBA may foster APP processing at multiple stages. Specifically, based upon molecular weight PBA appears to increase the α-secretase derived CTF-GV16 species. In order to determine the effects of PBA on γ-secretase, we examined PS1 and nicastrin levels. We observed no effect of PBA upon PS1 or nicastrin levels across the titrated concentrations (Figure 7B). Consequently, the stimulatory effects of PBA are not due to changes in the raw protein levels of the core γ-secretase components. Furthermore, PBA stimulated the accumulation of the CTF-GV16 proteolytic product in an additive manner with DAPT—wherein both PBA and DAPT elicit elevated levels of CTF-GV16, yet co-treatment of the NAG cells with both DAPT and PBA promoted increases in CTF-GV16 additively (Figure 7C). The additive effect suggests that PBA promotes APPGV16 cleavage by either α- or β-secretase.

### α- and γ-Secretase Mediated APP Cleavage Is Required for PBA Stimulation

**Figure 8:**
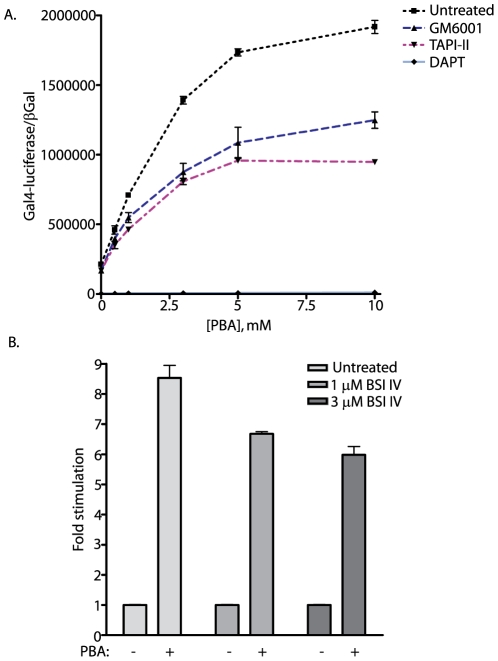
PBA stimulation of APPGV16 requires secretase processing. In order to determine whether PBA stimulation of APPGV16 is dependent upon proteolytic processing, NAG cells were treated with titrated levels of PBA in the presence or absence of specific secretase inhibitor:. 20 µM GM6001 (broad-spectrum metalloprotease inhibitor), 50 µM TAPI-II (α-secretase inhibitor), or 20 µM DAPT (γ-secretase inhibitor). PBA stimulated a statistically significant 9-fold increase in APP proteolysis (p<0.0001; student t-test). However, co-treatment with GM6001 or TAPI-II decreased the fold-stimulation by approximately half (p<0.0001; two-way ANOVA). DAPT significantly reduced the levels of activity and eliminated the stimulatory effect of PBA (p<0.0001; two-way ANOVA). The β-secretase contribution to PBA enhanced APP proteolysis was examined using two different concentrations of beta-secretase inhibitor (BSI) IV (B). PBA stimulated an 8–9 fold increase in normalized Gal4-luciferase activity. BSI IV decreased Gal4-luciferase activity by less than 25% (p<0.01; student t-test) (B). These data suggest that α-secretase plays a more significant role than β-secretase in PBA enhanced APP proteolysis.

In order to directly address which secretases are involved in PBA mediated stimulation of APP processing, the NAG cells were co-treated with PBA and pharmacological secretase inhibitors. In Figure 8A, NAG cells were treated with titrated levels of PBA in the presence or absence of either α-secretase inhibitors (GM6001 and TAPI-II) or high levels of the γ-secretase inhibitor DAPT. Consistent with previous observations, PBA alone stimulated dramatic changes in Gal4-luciferase output. However, treatment with either of the α-secretase inhibitors blocked approximately 50 percent of observed reporter output. Further, the PBA titration had no appreciable effect upon NAG cells pretreated with 20 µM DAPT (twice the concentration used in Figure 1). This strongly supports the requisite involvement of both the α- and γ-secretases in the PBA mediated stimulation of APP proteolysis. To examine the potential effects of PBA on β-secretase processing of APP, NAG cells were treated with PBA in the presence or absence of a β-secretase inhibitor (BSI IV). The data is normalized to fold-induction to quantify the observed effect. The application of either of two concentrations of BSI IV resulted in only a fractional repression of APPGV16 processing—approximately a 25 percent decrease from that observed in the absence of the β-secretase inhibitor (Figure 8B). These data indicate that PBA may impact both α- and β-secretase processing of APPGV16—with the stimulation of α-secretase contributing dominantly to the stimulatory effects observed with PBA.

### PBA Stimulates AICD Production in the Absence of Increased Amyloid Biogenesis

**Figure 9:**
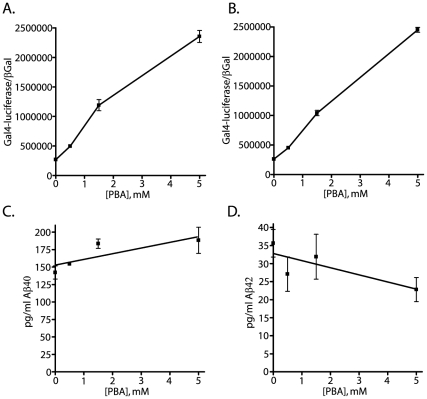
Amyloid biogenesis (Aβ40 and Aβ42) is unaffected by the PBA mediated stimulation in AICD production. The NAG cells were treated with titrated values of PBA (0, 0.5, 1.5, and 5 mM). A two-part assay measured γ-secretase dependent AICD production (A and B) and secreted amyloid biogenesis (C and D) from the same samples. The Gal4-reporter assays were performed with the cell lysate, while the media was assayed for species specific amyloid concentrations. PBA stimulated γ-secretase mediated proteolysis approximately ten-fold (A and B) in parallel assays to the ELISA measures for Aβ40 (C) and Aβ42 (D). Each concentration step in the PBA titration induced a statistically significant increase in Gal4-reporter activity ([PBA] shift: 0 to 0.5 mM, p<0.0003; 0.5 mM to 1.5 mM, p<0.0002; 1.5 mM to 5 mM, p<0.0001; analysis performed with the values in (A)). In contrast, there was no statistically significant change in either Aβ40 or Aβ42 levels. Aβ40 levels increased slightly from 142 picograms/ml to 188 picograms/ml, with a p-value of 0.09. In contrast, Aβ42 levels decreased from 35.6 picograms/ml to 22.7 picograms/ml, with a p-value of 0.06. In total, Aβ40 levels increased by 32.3 percent and Aβ42 levels decreased 36.05 percent—neither change reaching statistical significance. Each assay was performed in triplicate. The standard curves for Aβ40 and Aβ42 were linear in the tested concentration range with R^2^>0.96. Consequently, PBA stimulation of APP proteolysis occurs in a non-amyloidogenic manner.

The previous studies suggest that PBA has a minimal effect upon β-secretase mediated APP cleavage. However, to test the effects of PBA upon β-secretase processing directly, we performed parallel experiments examining AICD-GV16 induced Gal4-reporter activity and Aβ40 and Aβ42 production. The media was drawn off of the cells and employed in a classic sandwich ELISA procedure to measure the levels of Aβ40 and Aβ42 secreted during the PBA treatment period. After the media is drawn off and employed in the ELISA, the cells were washed, lysed and used to measure the levels of γ-secretase mediated AICD production by the reporter assays already discussed. By combining these two assays, we directly compared the effects of PBA on amyloid biogenesis and AICD generation. Separate sets of NAG cells were employed for the Aβ40 and Aβ42 assays, and accordingly, there are two separate assays for PBA effects upon AICD production. Consistent with previous experiments, PBA stimulated AICD production approximately 8–10 fold (Figure 9A and B). However, PBA had only minimal effects upon amyloid production. There is an approximately 32% increase in Aβ40 levels (142 pg/ml to 188 pg/ml) (Figure 9C) and a 35% decrease in Aβ42 levels (35.6 pg/ml to 22.8 pg/ml) (Figure 9D). The R^2^ value for each amyloid standard curve was at least 0.96 in all assays considered. All conditions were set-up and assayed in triplicate. The changes in Aβ40 and Aβ42 concentration across the PBA titration were not statistically significant at the p<0.05 level by either two-way ANOVA analysis nor by t-test comparisons of the high and low points of the curve. Repeated experiments consistently confirmed no significant effect of PBA on amyloid secretion levels. These data support a model in which PBA stimulates APP proteolysis through the promotion of α-/γ-cleavage.

### PBA Promotes AICD/FE65 Nuclear Signaling

**Figure 10:**
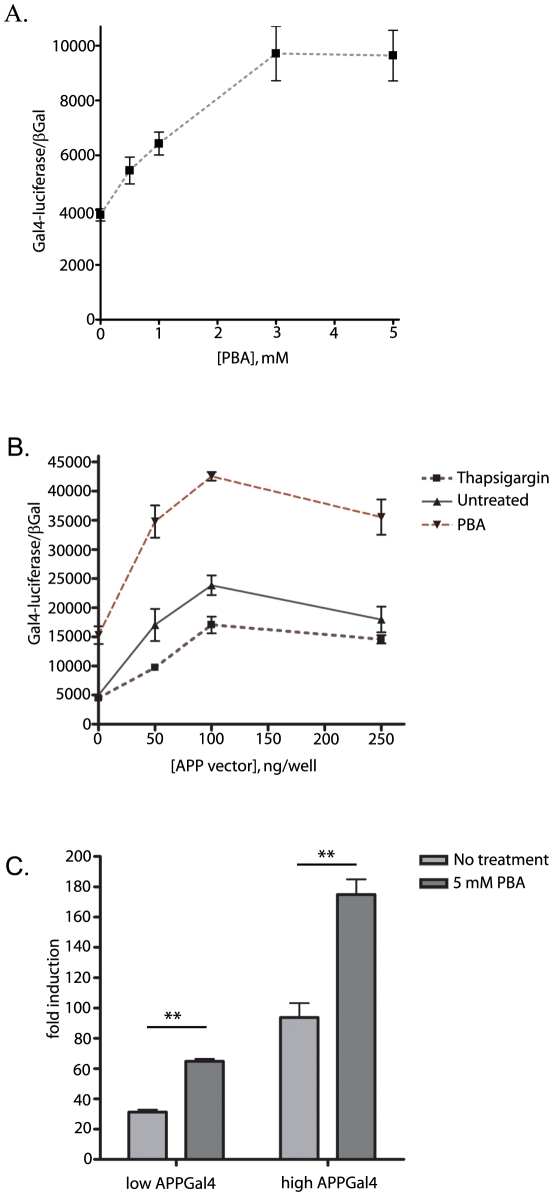
PBA stimulates APP/Fe65 nuclear signaling. Naïve N2a cells were transiently transfected with combinations of APP-HA and Fe65Gal4 (A and B) or with APP-Gal4 and Fe65 (C). Treatment with titrated PBA levels demonstrated that Fe65-Gal4 signaling significantly increases 2.5 fold with 5 mM PBA (p<0.005; student t-test of 0 and 5 mM PBA) (A). As maximal Fe65-Gal4 transcriptional signaling occurs at a specific APP:Fe65 ratio, N2a cells were transfected with titrated levels of APP. A 1∶1 vector ratio of APP:Fe65 stimulated maximal signaling in all treatment conditions. PBA significantly increased the Gal4-reporter activity over that observed with the untreated cells (p<0.0001; two-way ANOVA). Thapsigargin, in contrast, attenuated Gal4-reporter activity in comparison to the untreated cells (p<0.001; two-way ANOVA) (B). The APP-Gal4 assay was employed to test the consistency of the PBA mediated stimulation of AICD/Fe65 nuclear signaling. 24 hours post-transfection with APP-Gal4 and Fe65 at a 1∶1 vector ratio, the N2a cells were treated with 5 mM PBA for an additional 24 hours. Two vector concentrations were employed for these assays—low (50 ng) and high (500ng). At both vector concentrations, PBA elicited a significant 2-fold increase Gal4-luciferase activity (C) (p<0.005; student t-test).

Numerous genes may be regulated by APP nuclear signaling, in which the AICD, liberated from the membrane by γ-secretase cleavage, associates with Fe65 and travels to the nucleus to activate transcription [38], [39]. In order to address whether PBA or ER stress impact APP regulated gene expression, we employed two different Gal4 systems designed to assay AICD/Fe65 mediated reporter activation. The primary study employed Fe65-Gal4 [40] and HA-tagged APP, which were co-transfected at a 1∶1 ratio into naïve N2a cells with the Gal4-reporter and the EF1-βGal normalization vector. Subsequently, the transfected cells were treated with titrated concentrations of PBA for 24 hours and reporter activity was measured. PBA stimulated an approximate 250% increase in Fe65-Gal4 nuclear signaling (Figure 10A). In order to optimize the Fe65: APP ratio, naïve N2a cells were transfected with 100 ng/well Fe65-Gal4 and the quantity of APP expression vector was incrementally increased across transfection conditions. The cells transfected with titrated quantities of the APP expression vector were subject to either thapsigargin treatment, to induce ER stress, or PBA, to stimulate AICD production, or left untreated as the control. In all three treatment conditions, maximal activation of the Fe65-Gal4 reporter is observed when the two expression vectors are transfected at a 1∶1 ratio, which occurs at 100 ng/well of the APP expression vector (Figure 10B). Additionally, PBA stimulates Fe65-Gal4 signaling at statistically significant levels (p<0.0001; two-way ANOVA) above both the untreated and thapsigargin treated conditions across the APP vector titration (Figure 10B). The elevated level of Fe65-Gal4 induced reporter activity with PBA treatment, in the absence of any exogenously transfected APP, may be due to promotion of AICD production from endogenously expressed APP. Consistent with previous data showing thapsigargin induced decreases in APPGV16 cleavage, thapsigargin treatment of the APP/Fe65-Gal4 transfected cells resulted in a small, but statistically significant (p<0.001, two-way ANOVA), decrease in Fe65-Gal4 nuclear signaling relative to both untreated and PBA treated cells (Figure 10B).

In order to validate the stimulatory effect of PBA upon APP nuclear signaling, the APP-Gal4 [39] transcriptional activator was transfected into naïve N2a cells, along with Fe65, the Gal4-luciferase reporter, and the βGal normalization vector. Untagged Fe65 was co-transfected with APP-Gal4 at a 1∶1 vector ratio. To ensure that the reporter system was in a sensitive range, a low (50 ng) and high (500 ng) concentration of APP-Gal4 and Fe65 were used. At 24 hours post-transfection the cells were either left untreated, or stimulated with 5 mM PBA, for an additional 24 hours. In both low and high vector concentrations, PBA stimulated an approximate 2-fold increase in normalized reporter activity (Figure 10C). Interestingly, both reporter systems detected commensurate levels of increased APP nuclear signaling with PBA treatment, an approximate two-fold increase, which is considerably lower than the PBA mediated increase in AICD production. These observations support PBA induced stimulation of AICD production leading to elevated APP/Fe65 mediated nuclear signaling. Due to the disparity in level of PBA induced signaling between the APPGV16 assays and the AICD/Fe65 reporter assays, these data support a model in which γ-cleavage is not the only regulatory mechanism in APP nuclear signaling.

### PBA Blocks ER Stress-Induced Apoptosis

**Figure 11:**
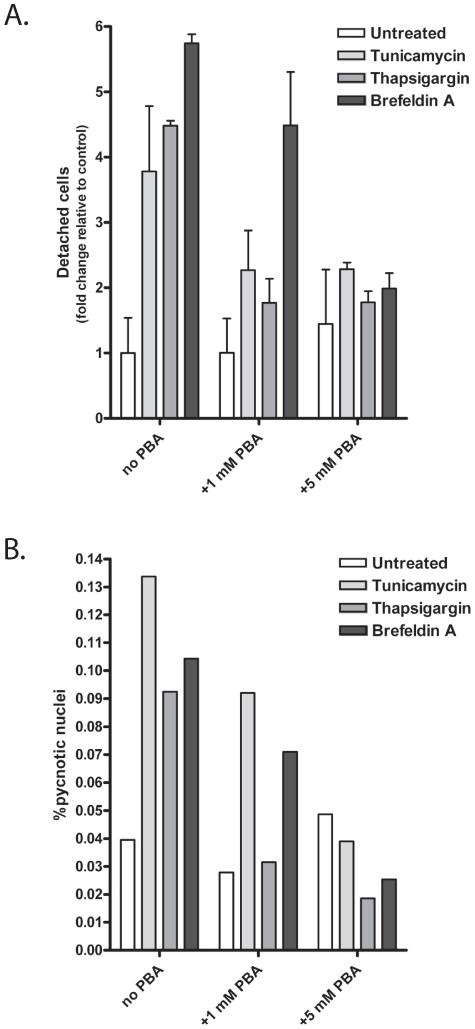
PBA decreases ER stress induced apoptosis. The cells were treated with vehicle control (1∶2000 DMSO) or the ER stress inducing agents: tunicamycin (5 µg/ml), thapsigargin (0.25 µM), or brefeldin A (5 µg/ml) in the presence or absence of PBA. A low and a high dose of PBA were used, 1 mM and 5 mM respectively, to assess concentration effects upon survival. The treatment was performed for 48 hours. After which, the detached cells were collected, spun down, and counted from triplicate plates on a standard hemocytometer. The number of detached cells increased by 3.8 fold (tunicamycin), 4.5 fold (thapsigargin), and 5.7 fold (BFA)—all of which were statistically different than basal levels (p<0.05) (A). With the addition of 1 mM PBA, the number of detached cells decreased to non-statistically significant levels in the tunicamycin and thapsigargin treated cells, while the numbers remained statistically different in the BFA treated cells. With 5 mM PBA co-treatment, the numbers of detached cells in all conditions were numerically and statistically indistinguishable from basal levels (A). The pycnotic nuclei were scored by an independent scientist blinded to the conditions. A minimum of 1000 cells were counted in each condition. The fraction of pycnotic nuclei are represented relative to the total number of cells counted (% of total). All three ER stress inducing agents elicited a two-three fold increase in pycnotic nuclei. The fraction of cells with pycnotic nuclei decreased with PBA treatment—in the thapsigargin treated cells, the number of pycnotic nuclei was identical to the untreated cells by 1 mM PBA, and by 5 mM PBA the fraction of cells with pycnotic nuclei reached basal levels in all three treatment groups (B).

Prolonged ER stress conditions are known to result in cellular apoptosis [13], [14]. Additionally, there are reports suggesting that Fe65/AICD nuclear signaling induces apoptosis [41], [42]. Consequently, the effects of ER stress induction and PBA stimulated AICD production upon cellular apoptosis were examined in NAG cells. The cells were either untreated or treated with a midrange dose of thapsigargin (0.5 µM), tunicamycin (1 µg/ml) or BFA (1 µg/ml) and co-treated with various concentrations of PBA from 0 to 5 mM. The cells were maintained for 48 hours under these conditions and then two separate assays of apoptosis were performed: a count of detached cells and a count of adherent cells with pycnotic nuclei. The detached cells were taken from three separate plates of treated cells. The numbers represent the totals summed from all sets of cells. In order to assure that there was no experimenter bias in the pycnotic nuclei count, the slides were coded and examined under blind conditions. Intriguingly, by neither assay did PBA alone stimulate any observable levels of apoptosis (Figure 11A and B). Additionally, PBA decreased the levels of apoptosis observed in thapsigargin, tunicamycin and BFA treated cells to levels near those of untreated cells within both assays. These data confirm the low toxicity and counter apoptotic effects of PBA reported by other groups, and suggest that elevation of the APP AICD alone is insufficient to induce measurable level of apoptosis, at least in the presence of PBA.

### Stimulation of AICD Production Is Not a Consistent Feature of Other Molecular Chaperones

**Figure 12:**
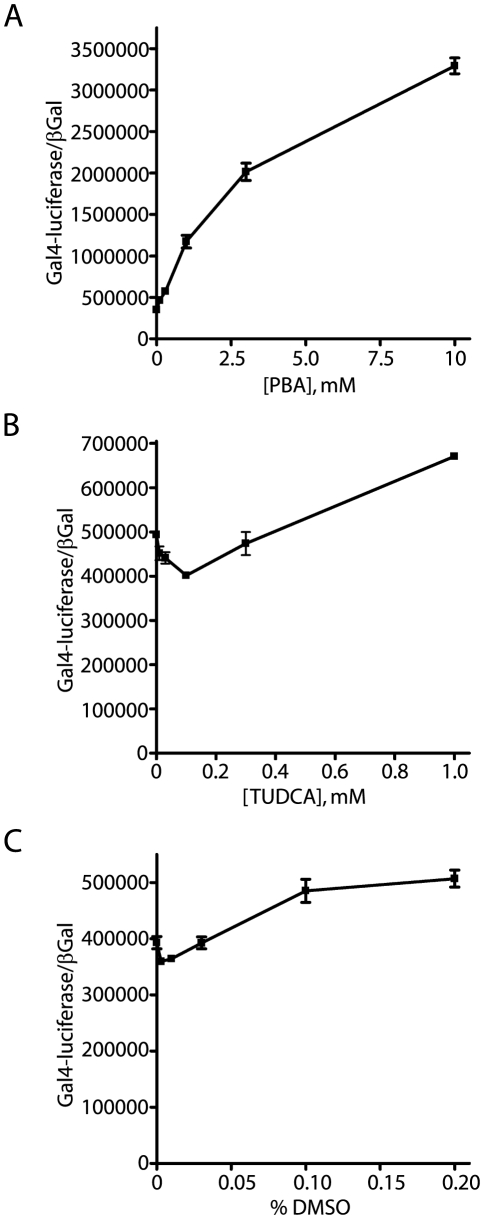
PBA specific effects upon APP proteolytic stimulation. In order to determine whether all small molecule chaperones (SMC) stimulate APP proteolysis, three different SMC were titrated onto NAG cells: PBA, TUDCA and DMSO. The concentration range for each titration was based on preceding experiments assaying the toxic levels of each compound. PBA stimulated a 9.3-fold increase in normalized reporter activity, which was statistically significant (p<0.0001, student t-test) (A). TUDCA elicited a statistically significant 1.35-fold increase in APPGV16 proteolysis (p<0.0001, student t-test) (B). DMSO treatment resulted in a smaller 1.29-fold increase, which was also statistically significant (p<0.003, student t-test) (C). All measures of statistical significance employed end-point analysis in which the untreated cells were compared to the final concentration in each titration. Each compound elicited a measureable increase in APPGV16 proteolysis, yet PBA stimulation was 6.89 times greater than TUDCA and 7.25 times greater than DMSO.

In order to assess the generality of molecular chaperone effects upon APP proteolytic processing, comparative titrations were performed with the NAG cells using three different small molecular chaperones: PBA, TUDCA, and DMSO. Consistent with previous experiments, PBA stimulated high levels of AICD-GV16 induced Gal4-reporter activity (Figure 12A). However, the PBA mediated effects upon APP proteolysis were not observed with either TUDCA or DMSO (Figure 12B and 12C). Both TUDCA and DMSO treatment elicited a 20–25% increase in observed proteolysis. Increasing the concentrations beyond the levels demonstrated in Figure 12 did not enhance the stimulatory capacity of any of the compounds, and resulted in high levels of toxicity with both TUDCA and DMSO. This indicates that not all members of the small molecular chaperone class are equivalently effective in stimulating APP proteolysis, and that PBA may have some of its physiological effects through other biochemical mechanisms.

## Discussion

The central question of this work is whether ER stress alters secretase mediated APP proteolysis. In order to address this question, we employed pharmacological treatment of the NAG cells, using agents known to alter protein processing within, or trafficking from, the ER. All pharmacological means of inducing ER stress result in the accumulation of misfolded proteins in the ER by distinct mechanisms. Our objective was to assess whether the general effects of ER stress alter APP processing; consequently, three different pharmacological stress inducing compounds are employed throughout this work—tunicamycin, thapsigargin, and BFA. Tunicamycin directly inhibits N-linked glycosylation of ER proteins, while thapsigargin targets SERCA-mediated calcium reuptake in the ER. Both of these approaches directly impact protein folding, as glycosylation-state promotes the transition from one conformational state to another—guiding the protein towards its native conformation [13], [14]. Similarly, blocking calcium reuptake into the ER impairs chaperone function—as many of the ER chaperones use calcium as a co-factor, and consequently depend on intralumenal calcium to direct proper protein folding [13], [14]. BFA directly targets the Arf-GTPase, and hence impairs ER to Golgi trafficking and promotes dissociation of the Golgi [33].

Our initial hypothesis purported that ER stress induced changes in trafficking would attenuate the levels of secretase mediated APP processing. In almost direct correspondence to UPR activation (Figure 2–4, blots) there was a partial arrest in APP proteolysis (Figures 2–4, bar graphs). As ER stress induction regulates both cellular transcriptional and translational responses, it could modify the reporter output associated with this assay system. However, there was no effect of ER stress induction upon βGal normalization or APPGV16 protein levels. Additionally, transiently transfected AICD-GV16, corresponding to the γ-secretase mediated APPGV16 cleavage product, produced stable activation of the Gal4-reporter when treated with thapsigargin, tunicamycin or BFA (Figure S2). Consequently, the decrement in APPGV16 driven reporter activity with ER stress induction, represents a decrease in proteolytic liberation of the AICD-GV16 moiety. Consistent with other work [7], [8], we find that induction of ER stress restricts APPGV16 localization to early components of the secretory pathway. As BFA blocks the trafficking of transmembrane proteins out of the ER, the similarity in APPGV16 localization pattern in tunicamycin and thapsigargin treated cells with BFA treated cells argues for a substantial ER localization in all three cases. The shift in subcellular localization could directly impact the association of APPGV16 with the mature secretases—arguing that ER stress responses may repress one or more stages of APP proteolytic processing.

The small molecular chaperone PBA alleviates ER stress and UPR signaling [35], [37]. Consistent with ER stress induction playing a primary role in the attenuation of APPGV16 proteolysis observed with all three stress inducing pharmacological agents—PBA rectifies the repression induced by tunicamycin and thapsigargin (Figure 5A). The putative mechanism of small molecular chaperones is the promotion of protein folding by decreasing the energy barrier between conformation states as the protein folds into its cognate conformation [25]. Consequently, PBA may rectify some portion of protein folding impaired by treatment with either tunicamycin or thapsigargin by facilitating a transition between conformation states in the absence of proper glycosylation or chaperone function. Increased protein folding would release APPGV16, and the cleaving secretase, from the ER retention and degradation processes employed to eliminate misfolded proteins[13]. However, there would be no rescue from stress related processes in cells treated with BFA, as ER localization is accomplished by targeting trafficking mechanisms which promotes Golgi dissociation[33]. The critical role of trafficking in APP proteolysis is highlighted by the complete lack of α- and β-cleavage products in the BFA treated cells (Figure 5B)—supporting other work demonstrating that secretase mediated cleavage occurs later in the biogenic pathway [9]. PBA mediated rectification of subcellular trafficking following tunicamycin and thapsigargin treatment (Figure 6), supports the direct relationship between APP trafficking and proteolytic regulation under ER stress conditions.

Intriguingly, PBA promotes APPGV16 trafficking (Figure 6) and dramatically stimulates secretase mediated APPGV16 cleavage (Figure 7) in the absence of any induced stress. The robust stimulatory effect of PBA upon APP proteolysis suggests that PBA may promote changes in the levels of APPGV16, the cleaving secretases, or their functional interaction. Yet, the change in APPGV16 proteolysis does not appear to be due to global changes in protein levels, as there was only a slight increase in APPGV16 and no changes in either Nicastrin or PS1 protein levels across the PBA titration (Figure 7B). The changes in APPGV16 protein levels observed are insufficient to account for the shift in reporter output—however, the CTFGV16 proteolytic species corresponding to the α-secretase cleavage product increased considerably across the PBA titration (Figure 7B). These data argue that overall protein synthetic rates are not the mechanism, but rather changes in the functional interactions between APP and the cleaving secretases are responsible for the proteolytic stimulation. This interpretation is consistent with the observation that PBA stimulates CTFGV16 production in the presence of DAPT (a well known γ-secretase inhibitor)—suggesting that PBA promotes an active association between APPGV16 and either the α- or β-secretases. This does not preclude PBA mediated stimulation of γ-secretase cleavage, as shifts in the subcellular localization of APPGV16 could alter proteolysis in the absence of change in γ-secretase protein levels.

PBA stimulation of α-secretase mediated APP cleavage is supported by experiments targeting either α- or β-secretase with selective inhibitors. Coordinate treatment with α-secretase inhibitors substantially repressed PBA mediated stimulation of APPGV16 cleavage (Figure 8A). The contribution of β-secretase to the PBA mediated stimulation of APPGV16 cleavage appears to be relatively minor—as the coordinate treatment of NAG cells with PBA and the β-secretase inhibitor BSI IV resulted in a relatively small decrease in the overall stimulation observed (Figure 8B). However, as the pathogenic Aβ42 form of amyloid is produced at considerably lower levels than Aβ40, relatively small changes in β/γ cleavage could result in relatively large shifts in amyloid ratios. Consequently, we examined the levels of each secreted amyloid species in direct comparison to reporter output. Consistent with the inhibitor studies, PBA had little effect upon the biogenesis of either amyloid species (Figure 9). These data suggest that PBA mediated stimulation of APP processing occurs predominantly through α/γ-cleavage, and does not significantly impact amyloidogenic processing. This is consistent with other work which suggests that under normal circumstances α- and β-secretase processing is not competitive [43]. Additionally, the NAG cells express sufficient levels of APPGV16 that competition for substrate between the α- and β-secretases may not occur. Furthermore, blocking γ-secretase activity with DAPT eliminates the PBA mediated enhancement of APPGV16 cleavage, demonstrating that the PBA mediated increases in reporter activity, are due to changes in the proteolytic processing of APPGV16.

Unlike amyloid secretion assays, the APPGV16/Gal4-reporter system interrogates the levels of AICD released into the intracellular compartment subsequent to γ-secretase mediated cleavage. The APP intracellular domain (AICD) forms a complex with Fe65, which is reported to traffic to the nucleus and activate gene expression following γ-secretase mediated APP cleavage [38], [39]. Numerous genes are implicated as APP/Fe65 regulatory targets including APP [44], neprilysin [45], KAI1 [38], GSK-3β [41] and others. While there is some contention about the validity and significance of these putative gene targets [46], we sought to assess whether PBA treatment or ER stress induction alters AICD/Fe65 nuclear signaling using recombinant reporter assays. Two separate assays were used: one in which Fe65 is fused to the Gal4 binding domain and co-transfected with wild-type human APP695 [47]; the other assay employs the Gal4 binding domain fused to the carboxy-terminus of APP695 [39]. Both assay systems demonstrated a significant increase in AICD nuclear signaling following PBA treatment, while stress induction by thapsigargin induced a repression in nuclear signaling (Figure 10A-C). Interestingly, PBA stimulation of Gal4-reporter activity via the transactivation potential of the AICD/Fe65 complex is far smaller than observed within the NAG cell proteolytic assay. This may be due to the far weaker transactivation capacity of the AICD/Fe65 complex relative to the potent GV16 transactivation domain. However, ER stress induction and PBA treatment act qualitatively similar upon AICD production (assayed with the APPGV16/Gal4-reporter assay) and AICD/Fe65 mediated Gal4-reporter activity—suggesting that repression of AICD production in ER stress and facilitation of AICD production with PBA treatment are both likely to alter AICD mediated gene expression.

One of the issues associated with pursuing α/γ-cleavage promoting agents as therapeutics in AD is the notion that either the AICD or the AICD/Fe65 complex may promote apoptosis. Numerous groups have reported neurotoxic effects associated with over-expression of the AICD [41], [42], [48], [49], [50]. Consequently, we examined the effects of prolonged PBA treatment in stressed and unstressed NAG cells. PBA treatment had no effects upon unstressed cells—suggesting that the elevated levels of AICD induced by PBA is insufficient to promote apoptosis (Figure 11A and B). In stressed cells, PBA prevented apoptosis in response to all three pharmacological treatments (Figure 11A and B). While these results are consistent with the anti-apoptotic effects of PBA in other systems [35], [51], it was surprising that PBA could overcome the apoptotic effects of BFA—as PBA has no effect upon the BFA induced repression of APP proteolysis. The butyrate short-chain fatty acid moiety of PBA inhibits histone deacetylation (HDAC) activity [52], which promotes neuronal survival [53]. The HDAC inhibitory capacity of PBA may contribute to its anti-apoptotic activity, potentially accounting for the apoptotic rescue observed in the BFA treated cells. The capacity of PBA to function as both a small molecule chaperone and an HDAC inhibitor may represent a convergence of biological activities that vests it with a unique therapeutic potential. Irrespective of the mode action underlying the anti-apoptotic effects of PBA, these data demonstrate that stimulation of AICD production alone is not guaranteed to induce apoptosis.

The potential multiplicity of biological roles of PBA led us to examine the effects of other members of the small molecular chaperone family upon APP proteolytic processing in the NAG cells. Two other commonly studied small molecules with reported chaperone-like function are taurine-conjugated ursodeoxycholic acid (TUDCA) and dimethylsulfoxide (DMSO) [13], [25], [37]. Neither DMSO nor TUDCA stimulated APPGV16 proteolysis analogously to PBA (Figure 12). The significance of these data is unclear—yet, one argument is that generic chaperone function is not sufficient to stimulate APP proteolysis, at least in the absence of ER stress inducing conditions. Alternatively, there may be some target specificity within the small molecular chaperone family through which different members are more effective in promoting the folding and trafficking of certain classes or types of proteins.

The plausibility of PBA providing therapeutic potential to AD patients is supported by a recent study in which the cognitive capacity of AD transgenic mice is rescued by transient PBA treatment [54]. Despite the cognitive rescue, the amyloid plaque pathology was not ameliorated. However, initiating PBA treatment in late stage pathological progression would not be expected to alleviate amyloid deposition as the plaques form prior to PBA treatment. Earlier administration of PBA may alter the relative abundance of α/γ and β/γ cleavage of APP. While we observe little effect of PBA upon amyloid biogenesis in vitro, PBA may have different effects in vivo than we observed within the NAG cells—most notably, the increase in α/γ-cleavage may drive down the levels of APP available to β-secretase. The lack of competition we observe within the NAG cells may be due to the profound over-expression of the APPGV16 substrate. In AD transgenic models in which ADAM10 is over-expressed, β-secretase mediated processing of APP decreases, amyloid plaque formation is lessened, and the cognitive capacities of the dual transgenic animals is improved [55]. Conversely, over-expression of the dominant negative ADAM10 leads to an exacerbation of the AD phenotype [56]. Taken together, these data suggest that the balance of α- and β-secretase mediated APP proteolysis may be a critical factor in determining the pathogenic progression in AD. Our group is currently examining the potential therapeutic effects of PBA in other AD transgenic mouse models, in which PBA administration begins prior to the pathological onset, and is administered across its anticipated progression.

The rationale for pursuing APP proteolytic stimulation via α/γ-cleavage is consistent with the growing body of evidence pointing to a loss of function associated with the genetically heritable Familial Alzheimer's disease (FAD) mutations [2]. Herein, the FAD mutations in PS1 are associated with a decrement in total amyloid levels [3] and a decrease in AICD production [4], [5]. As noted, the loss in γ-secretase proteolytic function may be mechanistically coupled with the increased production of the pathogenic Aβ42 amyloid species [57]. Pathogenic amyloid production may induce ER stress [58], [59], providing a mechanistic link between one of the cardinal features of AD and the manifestation of ER stress in AD patients. Deficits in proteolytic degradation and protein quality control observed in AD patients may promote protein aggregation [12], and subsequent ER stress induction, which could decrease γ-secretase mediated processing of APP in sporadic AD patients. In this context, the disruption of de novo protein maturation and trafficking in the ER may promote stress and UPR activation, and replicate the loss of function component of AD pathogenesis. The capacity of PBA to counteract ER stress and promote protein trafficking through the secretory pathway, along with PBA mediated stimulation of α/γ-cleavage, strongly supports the investigation into the therapeutic potential of PBA for the treatment of AD.

## Supporting Information

Figure S1 APPGV16 localization shifts to intracellular organelles with thapsigargin, tunicamycin or brefeldin A treatment. NAG cells were grown on 4 well slides to approximately 80% confluence and remained untreated (top row), or were treated with 0.25 µg/mL thapsigargin (Thaps, second row), 5 µg/mL tunicamycin (Tunic, third row), or 5 µg/mL brefeldin A (BFA, bottom row) for 18 hours. The cells were stained with the VP16 antibody (red) to determine localization of the APPGV16 protein. The cells were co-stained with Hoescht to label the nuclei (blue). In untreated cells, APPGV16 was detectable throughout the cell. In cells treated with thapsigargin or tunicamycin, the majority of APPGV16 was immediately adjacent to the nucleus, similar to the ER localization observed in the brefeldin A treated cells (BFA, bottom).
(11.11 MB TIF)

Figure S2 ER stress induction represses γ-secretase mediated cleavage of C99. The light bars represent N2a cells transfected with C99GV16 and the darker bars represent cells transfected with the γ-secretase cleavage product C50GV16 (AICD fused to GV16). The single star (*) denotes a p-value<0.05; while the double star (**) denotes a p-value<0.001. All three stress inducing compounds inhibit C99GV16 cleavage; yet only thapsigargin and brefeldin A repress proteolysis to a statistically significant degree. None of the stress inducing compounds had any repressive effect upon C50GV16, demonstrating that stress induction does not impair the function of the genetic reporter system.
(1.71 MB EPS)
