# Supplementary figures and images for: Phenylbutyric Acid Rescues Endoplasmic Reticulum Stress-Induced Suppression of APP Proteolysis and Prevents Apoptosis in Neuronal Cells
Source: PLoS One. 2010 Feb 9;5(2):e9135. doi: 10.1371/journal.pone.0009135 (PMC2817752; doi:10.1371/journal.pone.0009135)

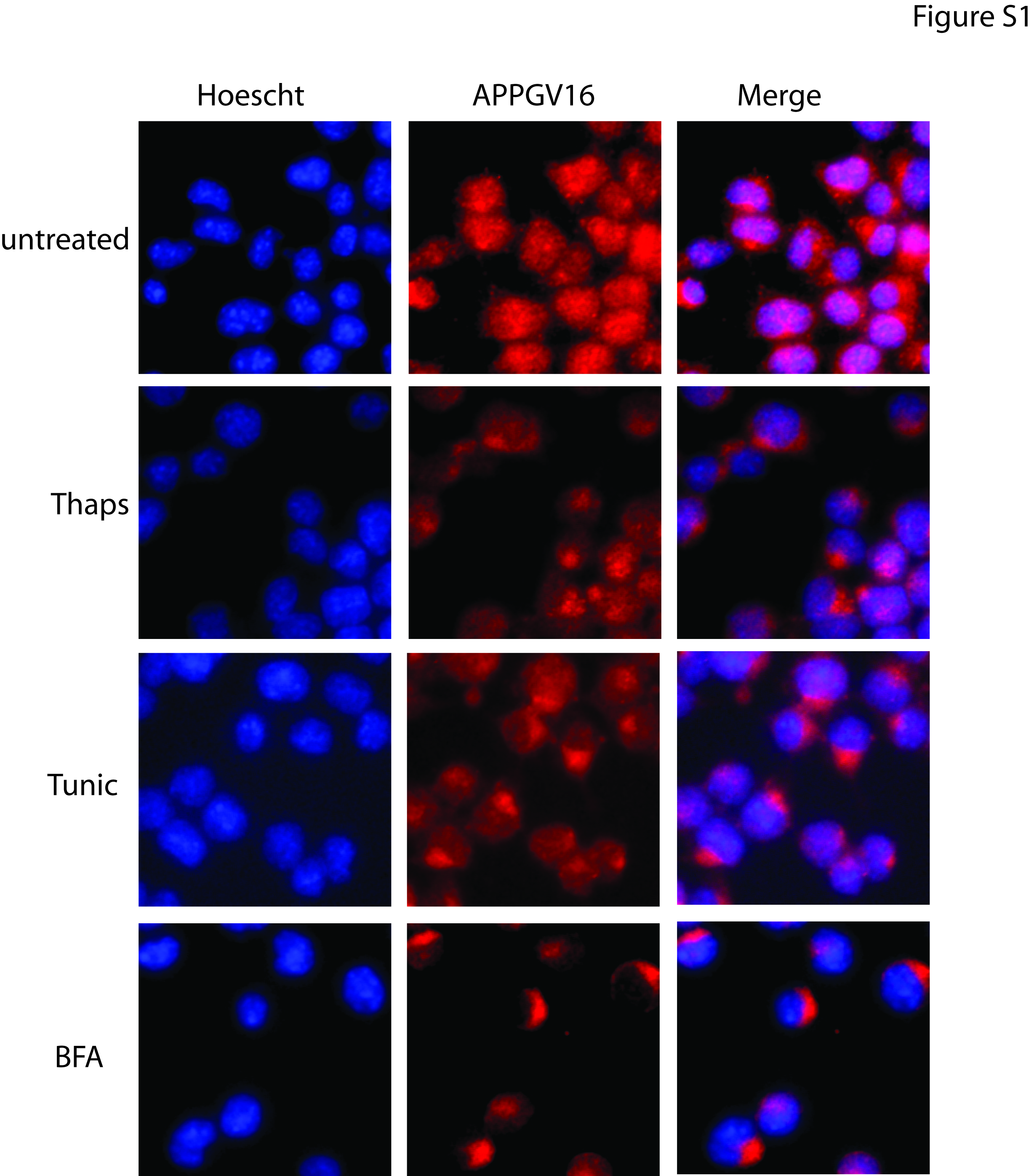

Supplement: Figure S1 — APPGV16 localization shifts to intracellular organelles with thapsigargin, tunicamycin or brefeldin A treatment. NAG cells were grown on 4 well slides to approximately 80% confluence and remained untreated (top row), or were treated with 0.25 µg/mL thapsigargin (Thaps, second row), 5 µg/mL tunicamycin (Tunic, third row), or 5 µg/mL brefeldin A (BFA, bottom row) for 18 hours. The cells were stained with the VP16 antibody (red) to determine localization of the APPGV16 protein. The cells were co-stained with Hoescht to label the nuclei (blue). In untreated cells, APPGV16 was detectable throughout the cell. In cells treated with thapsigargin or tunicamycin, the majority of APPGV16 was immediately adjacent to the nucleus, similar to the ER localization observed in the brefeldin A treated cells (BFA, bottom). (11.11 MB TIF) [file pone.0009135.s001.tif]
